# Performance of a large language model for identifying central line-associated bloodstream infections (CLABSI) using real clinical notes

**DOI:** 10.1017/ice.2024.164

**Published:** 2025-03

**Authors:** Guillermo Rodriguez-Nava, Goar Egoryan, Katherine E. Goodman, Daniel J. Morgan, Jorge L. Salinas

**Affiliations:** 1 Division of Infectious Diseases & Geographic Medicine, Department of Medicine, Stanford University School of Medicine, Stanford, CA, USA; 2 Division of Oncology, Department of Medicine, Stanford University School of Medicine, Stanford, CA, USA; 3 Department of Epidemiology and Public Health, University of Maryland School of Medicine, Baltimore, MD, USA; 4 University of Maryland Institute for Health Computing, Bethesda, MD, USA; 5 VA Maryland Healthcare System, Baltimore, MD, USA

## Abstract

We evaluated one of the first secure large language models approved for protected health information, for identifying central line-associated bloodstream infections (CLABSIs) using real clinical notes. Despite no pretraining, the model demonstrated rapid assessment and high sensitivity for CLABSI identification. Performance would improve with access to more patient data.

## Background

CLABSI significantly influences morbidity, mortality, and costs.^[1]^ CLABSI reporting is crucial for quality care in the United States.^[2]^ Most programs use active surveillance for CLABSI monitoring, with an infection control team reviewing inpatients based on National Healthcare Safety Network criteria to confirm true CLABSI events. However, subjective elements exist, including judging whether bacteremia is linked to a central line or originates from another source, and whether a skin commensal signifies infection or contamination.^[2,3]^ Computer algorithms designed to assist with this process have been explored, yet manual review remains standard.^[3]^

Large language models (LLMs) have emerged as advanced AI systems capable of processing coherent text and adapting to various tasks.^[4]^ They can perform tasks or predict outcomes for which they have not been explicitly trained. Some LLMs have passed medical exams^[7]^ and outperformed humans in evaluating clinical vignettes.^[5,7,8]^ However, standardized test questions and clinical vignettes have a concrete answer, whereas raw clinical documentation is complex and prone to subjectivity. In addition, due to privacy risks associated with AI chatbots,^[9]^ their performance with real patient information has been limited.

On January 29, 2024, Stanford Health Care and Stanford School of Medicine launched a secure LLM, powered by OpenAI’s GPT 4.0 (gpt-4-turbo), cleared for protected health information data. We aimed to assess this LLM’s performance in identifying CLABSI cases.

## Methods

**Figure 1.**
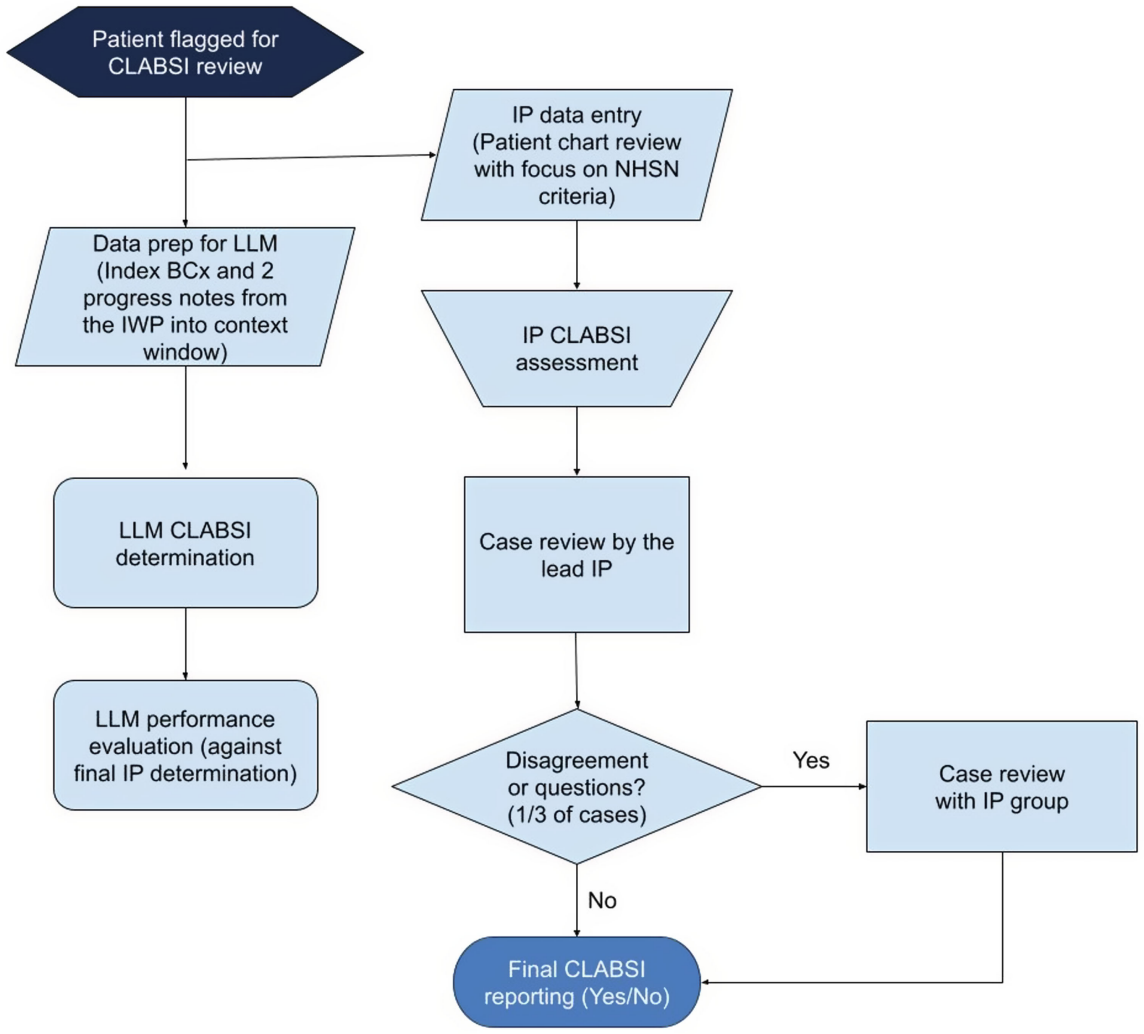
Comparative workflow of infection preventionists and LLM in CLABSI determination. The flow diagram illustrates the parallel workflows of IPs and an LLM. In the formal CLABSI review process, each case begins with an initial assessment by an IP after the patient is flagged by the Epic Bugsy™ EHR module for meeting the NHSN surveillance definition. This is followed by a thorough evaluation from the lead IP. If discrepancies or uncertainties arise, the case is escalated for further review by the infection prevention group, including the medical director or co-director, before a final determination is made. Abbreviations: CLABSI, central line-associated bloodstream infection; IP, infection preventionists; BCx, blood cultures; IWP, infection window period.

Stanford Health Care conducts active CLABSI surveillance, reviewing patients admitted for 48 hours with a central line in place for ≥3 days and a positive blood culture (Figure 1). Infection preventionists (IPs) use an Electronic Health Record (EHR) module (Epic Bugsy™) with full chart review capabilities to determine if an infection is a CLABSI or secondary bloodstream infection, based on NHSN criteria. Their findings are documented using a standardized NHSN abstraction form integrated into the EHR.^[10]^ For this pilot study, we selected 40 patients reviewed for CLABSI between November 2023 and March 2024: 20 consecutive CLABSI patients and 20 randomly sampled non-CLABSI patients. The low incidence of CLABSI made a larger sample impractical, and a smaller sample allowed quicker LLM assessment.

We prompted the secure LLM with clinical information and blood culture results, asking if each patient met the NHSN CLABSI definition. Due to token limits, we copied to the chat window only the blood culture results triggering the CLABSI alert and the last two progress notes within the infection window period (Table 1).

**Table 1.** Cases in which the LLM did not agree with IP assessment for CLABSI

Discordance reason (*N* = 17)	Breakdown	Discordance: LLM identified CLABSI not present per IP	Discordance: LLM missed CLABSI per IP
Missing information (*N* = 11)	Admission information would classify as community-onset BSI* (*N* = 4)	Patients with bloodstream infections present on admission documented in earlier notes or laboratory.	
	Missing cultures from other sources with matching organisms (*N* = 4)	Culture results from laboratory identified matching organisms including: – *E. faecium* adjudicated to cholecystitis from biliary drain cultures gram-positive cocci. – *C. parapsilosis* adjudicated to surgical wound infection. – *K. aerogenes* adjudicated to a groin wound infection. – *E. faecium* adjudicated to infected hemothorax.	
	Central line data and symptoms (*N* = 3)	– *C. albicans*, IP determined central line was not accessed before blood culture collection.	– IP determined CoNS CLABSI from fevers. – LLM did not identify symptoms or information regarding the central line. – In a case of *C. albicans* BSI and shock, the LLM reported having no information regarding the central line or symptoms.
Skin commensal or MBI organisms classified as CLABSI (*N* = 4)		– IP considered CoNS as commensal due to lack of symptoms, the LLM considered it a pathogen given the documentation of discitis-osteomyelitis. – IP considered *Streptococcus parasanguinis* commensal due to lack of symptoms, LLM identified fever. – IP considered *Fusobacterium nucleatum* an MBI organism, while the LLM considered it a CLABSI organism.^+^ – IP considered CoNS as commensal due to lack of symptoms, LLM identified fever and tachycardia.	
Pathogen considered skin commensal (*N* = 1)			*Brachybacterium* spp. bacteremia considered a skin commensal by LLM (1/4 bottles, lack of symptoms) IP noted NHSN does not list as commensal, and the patient had fever.
Alternative source (*N* = 1)			LLM considered *C. guilliermondii*, due to pancreatitis.

Abbreviations: LLM, large language model; IP, infection preventionist; CLABSI, central line-associated bloodstream infection; BSI, bloodstream infection; CoNS, coagulase-negative *Staphylococcus*; MBI, mucosal barrier injury; NHSN, National Healthcare Safety Network.

* Community onset: Blood cultures obtained within 2 days of admission.

+ NHSN guidelines list *Fusobacterium nucleatum* as an MBI organism.^[10]^

The LLM’s performance was quantified by comparing its determinations with the infection prevention group (the “gold standard”), calculating sensitivity, specificity, and agreement rates with 95% CIs.

## Results

From November 2023 to March 2024, the LLM identified 16 of 20 CLABSIs and 7 of 20 not CLABSIs. It achieved a sensitivity of 80% (95% CI 57.6%–92.9%) and a specificity of 35% (95% CI 33.3%–86.5%) for identifying CLABSI. The agreement rate between IPs and the LLM was 57.5% (95% CI 41.2%–73.3%).

The LLM’s limited data processing capacity impacted performance. Among 17 cases of disagreement between the LLM and the IPs, 11 (65%) were due to missing chart information (i.e., not in the most recent 2 progress notes or blood culture results). These included admission details (4 LLM false-positives: BSIs present on admission); matching BSI organisms with other clinical sources (4 LLM false-positives had other clinical cultures during the infection window period); and central line status or signs/symptoms, such as fever or shock (2 LLM false-negatives, 1 false-positive). When key information missing from the context window was included (Supplement), the LLM correctly changed its adjudication 10 out of 11 times, adjusting sensitivity to 90% (18/20), specificity to 75% (15/20), and agreement rate to 82.5%.

In the remaining discordant cases, the LLM misclassified a skin commensal or mucosal barrier injury organism as a true pathogen (4 false-positives), deemed a concurrent infection (pancreatitis) as the BSI source (1 false-negative), and misclassified a potential pathogen as a skin commensal (1 false-negative). Representative summaries of determinations are provided in the Supplement, and summaries of discordant cases are in Table 1.

Eight IPs reported a mean of 75 minutes (SD 48.7 minutes) to perform each CLABSI review. Furthermore, 77% reported reviewing over 5 clinical documents, including notes, flowsheets, medication records, and imaging studies, when evaluating CLABSI cases. The average time for LLM CLABSI review, including data inputting by the investigators, was 5 minutes for each case.

## Discussion

To our knowledge, this is the first U.S. study to evaluate the accuracy of an LLM in identifying CLABSI using real clinical notes. The LLM showed high sensitivity with limited data and no specific pretraining for CLABSI. While these results should be considered preliminary, they suggest that LLMs could eventually offer a promising “first-pass” screening tool for CLABSI detection by IPs, to winnow the pool of records requiring human review. The LLM’s data input and review process took 5 minutes, compared to 75 minutes per case for IPs.

Although the LLM demonstrated high sensitivity for identifying CLABSI, it had low specificity, resulting in many false-positives. This low specificity was due to practical limitations in the LLM’s implementation. Specifically, token limits (i.e., limits on the number of words that could be input) capped the data we could provide for review. Key details like admission information, other culture results or imaging studies and clinical information were often missing from the last two progress notes from the infection window period, causing discordance between IPs and the LLM. For example, four patients labeled as CLABSI by the LLM had BSI present on admission according to IPs. In three other cases, progress notes lacked culture results indicating a different source of bacteremia. With this additional information, the LLM’s sensitivity increased to 90% and specificity to 75%. Importantly, however, because IPs currently review all patients with bacteremia and central lines in place ≥3 days, even lower specificity in the context of high sensitivity could meaningfully reduce IP review workloads without sacrificing CLABSI identification.

In this pilot, we aimed to explore how an LLM could reduce the burden on IPs. We used full progress notes without curating them for relevant information. While this approach hindered results, it was more time efficient. An LLM integrated within the EHR and higher capacity to take more data would likely be more accurate and faster. Given the LLM’s goal to reduce IP burden, we believe this is the most operationally reasonable process.

Token limitations also restricted our ability to upload NHSN CLABSI guidelines for retrieval-augmented generation, which enhances LLM performance by providing reference materials. In one instance, the LLM classified *Brachybacterium* spp. as a skin commensal, whereas the NHSN guidelines do not recognize it as such.^[10]^ Additionally, context retention issues, where the LLM could not maintain relevant details across interactions in the same context window, prevented consistent application of learned information.

Finally, while we evaluated the LLM’s performance in strictly applying NHSN criteria for CLABSI as a binary outcome, some of its inaccurate conclusions were often reasonable from a clinical perspective. For example, the IP deemed coagulase-negative *Staphylococcus* as commensal based on NHSN criteria, lacking definitive symptoms. In contrast, the LLM flagged it as pathogenic, interpreting a temperature of 37.8°C (100.2°F) coupled with tachycardia as indicative of fever.

In conclusion, this secure LLM powered by OpenAI’s GPT-4 demonstrated high sensitivity for identifying CLABSI from real clinical notes. We expect its specificity to improve with access to more data from patient records and training using NHSN documents and sample cases. Our experience provides insights for institutions that may soon implement LLM technologies, including significant potential efficiency improvements but also practical challenges that can limit performance. We view LLMs as a promising tool to assist, not replace, human CLABSI review.

## Supporting information

Rodriguez-Nava et al. supplementary material Rodriguez-Nava et al. supplementary material

## Supplementary material

The supplementary material for this article can be found at https://doi.org/10.1017/ice.2024.164.
