# Supplementary material for: Performance of a large language model for identifying central line-associated bloodstream infections (CLABSI) using real clinical notes
Source: Infect Control Hosp Epidemiol. 2024 Oct 30;46(3):305–8. doi: 10.1017/ice.2024.164 (PMC11883649; doi:10.1017/ice.2024.164)
Supplement: Rodriguez-Nava et al. supplementary material [file S0899823X24001648sup001.docx]

**Supplementary Table 1. Summary of CLABSI Determinations Based on IP and LLM Assessments.**

| Categorization | Rationale/Reasoning |
| --- | --- |
|  |  |
| CLABSI by IP and LLM  (True positive) | **IP:** A patient with myelodysplastic syndrome admitted for allogeneic stem cell transplant. Post-transplant complicated by hematuria, febrile neutropenia, bacteremia, and hypotension along and elevated troponin levels. Blood cultures on hospital day 17 revealed CoNS in 4 bottles and on hospital day 20 and *Clostridium ramosum* and *Gemella* spp. Central line from admission to hospital day 21. Hypotension on hospital day 20 requiring dialysis and transfer to the MICU. The patient met LCBI 2 criteria due to hypotension and CoNS in all 4 bottles. Cannot be MBI due to 2 matching common commensals.  **Secure LLM:** The patient does meet the NHSN definition for CLABSI. Rationale: The definition of CLABSI according to NHSN involves the isolation of a pathogen from a blood culture in a patient who had a central line at the time of or within 48 hours preceding infection and who did not have an infection at another site. Considering these new data: The patient had a central line at the time of infection. The blood culture report shows the presence of CoNS, repeated in two different times, which considers it a pathogen rather than a contaminant. All other potential sources of infection need to be ruled out (the patient does not seem to have an infection at another site based on the provided notes). |
| CLABSI by IP and LLM  (True positive) | **IP**: The patient was admitted to bone marrow transplant unit. The patient developed febrile neutropenia. Blood cultures conducted on 12/31/23 showed all 4 out of 4 samples positive for CoNS, with pending susceptibilities. Additionally, blood cultures conducted on hospital day 19 blood cultures with 1 out of 4 bottles with anaerobic *Gemella* and 2 out of 4 bottles with *Streptococcus viridians*, but no further identification was sent. The patient also had skin lesions, including one on the lower extremity with a "folliculitis" appearance, although did not meet the NHSN definition for soft tissue and skin infection.  **Secure LLM**: The patient could be meeting the NHSN definition for a CLABSI. The factors contributing to this conclusion are: The patient has a history of CoNS bacteremia and CoNS have been again identified in blood cultures (as per the medical data provided previously). The patient on day +18 post-transplant has neutropenic fever without another recognized cause and has been on broad-spectrum antibiotics. The patient had a PICC line associated with previous DVT, and the line was in use. Notably, as per the records, the patient had CoNS bacteremia/CLABSI in almost a year back, suggesting that the line is a possible source of the current blood infections. Moreover, the PICC line was recently removed due to persistent bacteremia, which is typically a strategy when CLABSI is suspected. |
| Not CLABSI by IP and LLM  (True negative) | **IP**: The patient was admitted due to abdominal discomfort. An abdominal MRI indicates worsening third spacing including small to moderate volume ascites and anasarca. On hospital day 28 an MRI showed gastric and colonic wall edema may be due to third spacing or infection. On hospital day 33 had blood cultures with *Candida glabarata*. Infectious disease suggests the possibility of enterocolitis and continuation of Meropenem for treatment. Given that the candidemia is most likely due to GI translocation from ischemic colitis, with caspofungin identified as an appropriate treatment, meets criteria for gastrointestinal tract condition.  **Secure LLM:** The patient does not fully meet the NHSN definition of a CLABSI. One of the main criteria is the presence of a central line or umbilical catheter at the time of, or within 48-hours prior to, the onset of symptoms. There is no mention of central line placement in the available information. Additionally, although we have evidence of *Candida glabrata* in a blood culture (which is a common skin contaminant), we don't have a matching pair of blood cultures drawn on separate occasions which is required when a common skin contaminant is identified. Fulfilling this criterion along with a proper symptom manifestation (fever, chills, or hypotension) would satisfy the second part of the NHSN's CLABSI definition. Although the patient indeed has *Candida* in the blood, we only have one set of blood cultures and one single instance, so this alone does not meet the NHSN definition of a CLABSI. |
| Not CLABSI by IP and LLM  (True negative) | **IP:** Blood cultures positive with *Clostridium paraputrificum* before colorectal surgery. Adjudicated to surgery.  **Secure LLM**: The patient does not meet the NHSN definition for CLABSI. NHSN criteria require that the patient have a recognized pathogen cultured from one or more blood cultures and the organism cultured from blood is not related to an infection at another site. In the provided scenario, *C. paraputrificum* was detected, which is mostly associated with intra-abdominal infections. The patient had recent bowel ischemia and a surgery, thus it appears this bacteremia could be secondary to his abdominal condition rather than a primary bloodstream infection resulting from a central line. |
| CLABSI by LLM but not IP  (False positive) | **IP:** A patient with interstitial lung disease was admitted for bilateral lung transplant, complicated by right ventricular dysfunction, MDR *Escherichia coli* pneumonia, and E. faecium bacteremia secondary to acalculous cholecystitis. On hospital day 46 developed hypotension. Blood cultures with *E. faecium* in 2 out of 2 bottles. Cholescintigraphy scan on hospital day 48 with evidence suggestive of cholecystitis. IR-guided drain placed on hospital day 49 with fluid growing gram-positive cocci.  **Secure LLM:** The patient does meet the NHSN criteria for CLABSI. Here is the rationale: The patient has an *E. faecium* identified from blood culture, which is a pathogen typically associated with CLABSI. The patient had a central line during the onset of infection, as noted in progress notes "...RIJ and right femoral ECMO cannula...", which were in place before the development of the bloodstream infection. The patient appears to have symptoms of associated infection including reported "MDR *E. coli* pneumonia." |
| CLABSI by LLM but not IP  (False positive) | **IP:** A patient was transferred for ventricular tachycardia storm and was treated with right and left ventricular assist devices. Vascular access included hemodialysis triple line, pulmonary artery catheter, and midline catheter. On hospital day 52, a blood cultures grew *Klebsiella aerogenes*. The left groin wound/incision was also cultured due to warmth and erythema on hospital day 54, growing the same organism. The patient meets criteria for skin and soft tissue infection with secondary bloodstream infection caused by *K. aerogenes.*  **Secure LLM**: The patient seems to have a CLABSI. Reasoning: The patient's blood cultures are positive for a pathogenic organism, carbapenem-resistant *K. aerogenes*. The same organism is found in a wound culture that's likely non-related to the central line site. There's mention of hemodynamic supports like left and right ventricular assist devices, dobutamine - suggestive of central line usage. The patient presented various symptoms over this course of care, including temperature changes and hypotension, as mentioned in the context. |
| CLABSI by IP but not LLM  (False negative) | **IP:** A patient with history of lumbar abscess and MSSA purulent pericardial effusion was admitted for aortic pseudoaneurysm requiring total arch replacement and ascending aorta replacement. The patient experienced complications including septic shock, MSSA mediastinitis/aortitis, ESBL *E. coli* pneumonia/empyema, *Candida* empyema, tracheal perforation, and esophageal perforation. The patient was transferred for cardiothoracic surgery evaluation and had a tracheal stent placed. On hospital day 10, fever was noted, and blood culture collected, which grew *Candida albicans*. The patient had concurrent *Candida* empyema, but no new cultures were collected during infection window period.  **Secure LLM:** The patient had a history of multiple complex medical issues including MSSA bacteremia among others, but it does not indicate the presence of a CLABSI at this time. The CDC/NHSN criteria for a LCBI – associated with central line, requires the patient to have a recognized pathogen cultured from one or more blood samples and the organism cultured from blood is not related to an infection at another site. Though *C. albicans* was identified in the previously provided blood culture results, it is not explicitly stated that this candidemia is related to a central line infection in the provided patient records. Additionally, no information about the patient’s central line status, nor any new symptoms, signs, or related treatments indicative of a potential CLABSI are mentioned in these reports. Therefore, based on the information provided, the patient does not meet the NHSN definition for CLABSI at this time. |
| CLABSI by IP but not LLM  (False negative) | **IP:** A patient was admitted for a myeloablative allo-transplant. The cell culture detected *Rosemonas* but was deemed a likely contaminant. Meropenem was administered empirically, and cell transfusion was without incident. A PICC was in place before admission. On hospital day 7, blood cultures grew *Brachybacterium* spp. in 1 bottle. The patient developed a fever of 38.7 C on hospital day 10. CT chest, abdomen, and pelvis outside the infection window period and no chest X-rays during the infection-window period to attribute to pneumonia. The patient meets LCBI-1 criteria.  **Secure LLM:** The patient doesn't meet the NHSN definition for CLABSI. Here's why: The definition of CLABSI according to NHSN is: “A laboratory confirmed bloodstream infection where central line or umbilical catheter was in place for >2 calendar days on the date of event, with the day of device placement being Day 1, and the central line or umbilical line was in place on the date of event or the day before.” In the provided information, the patient does show signs of infection, such as the blood culture being positive with *Brachybacterium* spp. However, it’s important to note that *Brachybacterium* spp. is often considered a skin contaminant and one positive bottle on blood culture is usually not sufficient to diagnose a bloodstream infection. Furthermore, the patient is reported to be afebrile (does not have a fever) and there is no specific mention of symptoms that could be connected to a bloodstream infection such as chills or hypotension. |

Abbreviations: CLABSI, central line-associated bloodstream infection; IP, infection preventionists; LLM, large language model; CoNS, coagulase-negative *Staphylococcus*; MICU, medical intensive care unit; LCBI, laboratory-confirmed bloodstream infection; MBI, mucosal barrier injury; NHSN, National Healthcare Safety Network; PICC, peripherally inserted central catheter; DVT, deep venous thrombosis; MDR, multi-drug resistant; RIJ, right internal jugular; ECMO, extracorporeal membrane oxygenation; MSSA, methicillin-susceptible *Staphylococcus aureus*. ESBL, extended spectrum beta-lactamase.

One of two investigators (GRN and GE) prompted the secure LLM with the instruction: “*I will provide clinical information and blood culture results. Based on this information, tell me if the patient meets the NHSN definition for CLABSI. Limit your answers to yes or no and explain the rationale.*” They then copy-pasted (1) the blood culture results that triggered the CLABSI alert; and (2) the last two progress notes written by the primary care team during the infection window period (defined by the NHSN as the 3 days before and 3 days after the first positive test) [10]. We restricted the data entered to two notes because of token limitations (the maximum amount of text an LLM can analyze at once). Stanford Health Care has set it at 128,000 tokens with an output limit of 4096 tokens. We selected the last two notes from the infection window period, because we considered them to most likely to contain all necessary details to attribute the bloodstream infection to other sources, if any. Each case was inputted in a new context window.

**Supplementary Table 2. Revised CLABSI Determinations Incorporating Additional Data**

| Patient ID | Previous GPT Determination | Previous GPT Rationale | IP Determination | IP Rationale | Organism | Reason for Disagreement | Added Info | New GPT Determination | GPT Updated Response |
| --- | --- | --- | --- | --- | --- | --- | --- | --- | --- |
| 8 | No | No clear linkage of bacteremia to central line or umbilical catheter use within the previous 48 hours and absence of accompanying symptoms like fever, chills, or hypotension. | Yes | PICC line in place and removed; Meets LCBI 2 criteria due to common commensal and fevers. | Coagulase-*negative Staphylococcus* | LLM failed to capture central line information and symptoms. | Clinical data | Yes | Yes, the patient meets the NHSN definition for CLABSI. The blood cultures were positive with CoNS, where all 4 bottles from the drawn blood culture tested positive for CoNS. Additional clinical notes indicate the patient had a persistent fever and was on an antibiotic regimen, which shows signs of an ongoing infection. |
| 19 | No | Patient history and symptoms do not indicate the presence of CLABSI; *Candida albicans* in blood culture is not explicitly linked to a central line infection. | Yes | Patient had a PICC line and tunneled dialysis femoral line; Fever and positive blood culture for *Candida albicans*. | *Candida albicans* | LLM failed to capture central line information and symptoms. | Clinical data | Yes | Patient has PICC line and blood culture showing *Candida albicans*, aligning with NHSN criteria assuming the central line has been in place for over 2 days. |
| 26 | Yes | Meets NHSN definition for CLABSI with clinical history, detection of *Enterococcus faecium* in blood culture, absence of secondary infection source; requires additional clinical information. | No | Multiple cultures sent during thoracotomy and noted purulence. Identified *E. faecium* from thoracotomy fluid culture, consistent with secondary BSI. | *Enterococcus faecium* | LLM failed to capture thoracotomy fluid culture organism. | Thoracotomy cultures | No | From the clinical notes provided, no, the patient does not meet the NHSN definition for CLABSI in this instance. Rationale: The specimen isolated the presence of Enterococcus faecalis and CoNS from the lung tissue biopsy. This indicates that the infection source is likely from the lung (possible pneumonia), and not the central line. |
| 27 | Yes | Central line in place, positive for *Staphylococcus aureus* in blood cultures, hypotension requiring vasopressor support. | No | Positive blood culture indicating community onset MSSA. | *Staphylococcus aureus* | LLM failed to recognize this was present on admission. | Admission date | No | Thank you for the additional information. If the positive blood culture for *Staphylococcus aureus* was obtained on the day of admission, which is before the central line was placed, this would not meet the criteria for a CLABSI. |
| 28 | Yes | Identified *Enterococcus faecium* from blood culture with associated infection symptoms; central line present. | No | *E. faecium* isolated from gallbladder fluid culture secondary to acute cholecystitis; matching blood and gallbladder fluid cultures indicate secondary BSI. | *Enterococcus faecium* | LLM failed to identify gallbladder fluid culture organism. | Biliary cultures | No | Based on this provided information, the patient does NOT meet the NHSN definition for CLABSI. This statement is supported by the fact that an infection is isolated from the gallbladder fluid culture, indicating that the bacteremia could be secondary to the acute cholecystitis. |
| 29 | Yes | Central line devices in place; blood culture positive for *Candida parapsilosis* complex; absence of alternative infection source. | No | Matching organism in blood and neck wound cultures, consistent with secondary BSI. | *Candida parapsilosis* complex | LLM failed to recognize wound culture organism. | Wound cultures | No | Given this additional information, the positive blood culture for *Candida parapsilosi*s can be associated with the infection at another site, which is the surgical wound on the right neck. |
| 30 | Yes | Central line placement, positive blood culture for Candida albicans, symptoms consistent with infection. | No | Patient's single lumen port not accessed; positive blood culture from peripheral line before central line access. | *Candida albicans* | LLM failed to capture line access details. | Urine cultures | No | Based on the provided information, the patient does seem to meet the NHSN definition for CLABSI. If additional cultures (such as the urine culture you posted) are also positive for Candida albicans, this could be suggestive of a common source of infection and would possibly complicate the diagnosis of CLABSI. The source of the yeast may not be the central line but could be related to an urinary tract infection, especially if there was an indwelling urinary catheter. |
| 33 | Yes | Meets NHSN criteria for CLABSI: Enterococcus faecalis and CoNS presence in blood cultures, central line present at the time. | No | Positive blood cultures on admission indicate POA with RIT definition. | *Enterococcus faecium* | LLM failed to recognize this was present on admission and RIT. | Admission date | Yes | Yes, the patient does meet the definition for CLABSI. This conclusion is based on the positive blood culture for *Enterococcus faecalis*, a known pathogen. There is no other information provided that suggests an alternative source for the bacteremia. The clinical notes suggest that the patient had a central line in place at the time of or prior to culture collection. |
| 34 | Yes | Meets NHSN CLABSI criteria: bloodstream infection with Klebsiella aerogenes, no alternative infection source, central lines in use. | No | Matching organism in wound and blood cultures indicating secondary BSI. | Klebsiella aerogenes | LLM failed to capture matching skin organism. | Skin culture | No | Based on the provided information, the answer will be No. The patient does not meet the NHSN definition for a CLABSI. The patient has the pathogen, *Klebsiella aerogenes*, in both the wound culture and the blood culture. This raises the possibility that the blood infection could be due to a potentially infected wound, rather than a central line. |
| 38 | Yes | Meets NHSN definition of CLABSI: central line in place for >2 days, positive blood cultures with CoNS, patient symptomatic with fever. | No | Bloodstream infection present on admission. | Coagulase-negative *Staphylococcus* | LLM failed to recognize this was present on admission. | Admission date | No | Thanks for the clarification on dates. However, under NHSN guidelines, a bloodstream infection isn't considered a CLABSI if the blood culture is drawn on the day of or the day after central line insertion. Since the cultures were obtained one day after admission (which is presumably when the central line was inserted), this case wouldn't meet the NHSN definition for a CLABSI. |
| 40 | Yes | Yes, patient meets NHSN criteria for CLABSI: *Candida glabrata* in bloodstream, inferred central line usage due to symptoms of infection and no alternative source. | No | BSI present on admission. | *Candida glabrata* | LLM failed to recognize this was present on admission. | Admission date and unit | Yes | Appreciate your clarification. The PACU stay is typically brief and used for recovery immediately after surgery. The bloodstream infection with yeast was reported more than 48 hours after the admission and therefore not considered as a community-acquired infection. However, without explicit details on when a central line may have been placed and whether it was in place for over two calendar days during this hospital stay, I can't definitively state if it meets the NHSN definition for a CLABSI |

Abbreviations: CLABSI, central line-associated bloodstream infection; PICC, peripherally inserted central catheter; LCBI, laboratory-confirmed bloodstream infection; LLM, large language model; IP, infection preventionists; NHSN, National Healthcare Safety Network; CoNS, coagulase-negative *Staphylococcus*; MSSA, methicillin-susceptible *Staphylococcus aureus*; BSI, bloodstream infection.
